# Long-Term Visuo-Gustatory Appetitive and Aversive Conditioning Potentiate Human Visual Evoked Potentials

**DOI:** 10.3389/fnhum.2017.00467

**Published:** 2017-09-21

**Authors:** Gert R. J. Christoffersen, Jakob L. Laugesen, Per Møller, Wender L. P. Bredie, Todd R. Schachtman, Christina Liljendahl, Ida Viemose

**Affiliations:** ^1^Department of Food Science, University of Copenhagen Frederiksberg, Denmark; ^2^Department of Biology, University of Southern Denmark Odense, Denmark; ^3^Department of Psychological Sciences, University of Missouri Columbia, MO, United States

**Keywords:** human brain, EEG, visual evoked potentials, conditioning, gustatory

## Abstract

Human recognition of foods and beverages are often based on visual cues associated with flavors. The dynamics of neurophysiological plasticity related to acquisition of such long-term associations has only recently become the target of investigation. In the present work, the effects of appetitive and aversive visuo-gustatory conditioning were studied with high density EEG-recordings focusing on late components in the visual evoked potentials (VEPs), specifically the N2-P3 waves. Unfamiliar images were paired with either a pleasant or an unpleasant juice and VEPs evoked by the images were compared before and 1 day after the pairings. In electrodes located over posterior visual cortex areas, the following changes were observed after conditioning: the amplitude from the N2-peak to the P3-peak increased and the N2 peak delay was reduced. The percentage increase of N2-to-P3 amplitudes was asymmetrically distributed over the posterior hemispheres despite the fact that the images were bilaterally symmetrical across the two visual hemifields. The percentage increases of N2-to-P3 amplitudes in each experimental subject correlated with the subject’s evaluation of positive or negative hedonic valences of the two juices. The results from 118 scalp electrodes gave surface maps of theta power distributions showing increased power over posterior visual areas after the pairings. Source current distributions calculated from swLORETA revealed that visual evoked currents rose as a result of conditioning in five cortical regions—from primary visual areas and into the inferior temporal gyrus (ITG). These learning-induced changes were seen after both appetitive and aversive training while a sham trained control group showed no changes. It is concluded that long-term visuo-gustatory conditioning potentiated the N2-P3 complex, and it is suggested that the changes are regulated by the perceived hedonic valence of the US.

## Introduction

Identification and selection of food items or beverages is often based on their visual appearance. This is made possible because the visual stimulus serves as cue for recall of long-term stored associations between previously seen ingested substances and their flavor. The allocation of visuo-gustatory combinations to explicit memory is one that begins early during infancy and is refined throughout life. In spite of the importance of such associations, only one previous publication has reported on human brain electrophysiological plasticity related to such long-term conditioning (Viemose et al., 2013). However, numerous EEG-studies have analyzed associations between visual conditioned stimuli (CSs) and non-gustatory unconditioned stimuli (USs) and have found that visual evoked potentials (VEPs) can be modified by classical conditioning. For instance, visual stimuli have been paired with auditory USs: the sight of an arrow (CS) combined with high intensity clicks (US) increased the amplitudes of a negative CS-induced VEP-wave with a peak delay of approximately 155 ms (Begleiter and Platz, 1969b). Pairings of faces with a loud aversive noise resulted in stronger face-induced currents compared to unpaired faces in the inferior temporal and the fusiform gyri containing the “fusiform face area” (Sergent et al., 1992; Kanwisher et al., 1997; Parvizi et al., 2012), as well as in cuneus and precuneus (Pizzagalli et al., 2003).

Visual CSs have also been paired with visual USs: neutral nonsense trigrams (CSs) paired with pictures (USs) of emotionally arousing capacity (Johnston et al., 1986) resulted in the increase of a positive VEP-wave (P4, peak delay between 540 ms and 660 ms). This type of association contained both predictive and evaluative conditioning (Staats and Staats, 1958; Baeyens et al., 1993, 2001; Rozin et al., 1998; Field, 2000; De Houwer et al., 2001), and the fact that the affective associations changed a late positive component is in agreement with reports documenting that affective evaluations are reflected in the amplitude of the late part of VEPs (Johnston et al., 1986; Mini et al., 1996; Palomba et al., 1997).

Visual nonsense words paired with painful shocks lead to enhanced amplitudes of an N100 peak evoked by paired but not unpaired CSs (Montoya et al., 1996). Late slow components (400–800 ms after stimulus onset) were also affected, suggesting that the aversive effect of the painful US was transferred to the nonsense words through evaluative conditioning, since late components are sensitive to the affective value of photically presented words (Begleiter and Platz, 1969a; Naumann et al., 1992). Other investigations have paired faces (Flor et al., 1996), Landolt rings (Skrandies and Jedynak, 2000) or black and white grating patterns (Baas et al., 2002) with electric stimulations and have documented plasticity in all phases of VEPs from the earliest to the latest among the “endogenous” components (Näätänen and Gaillard, 1983; Okada et al., 1983; Stapleton and Halgren, 1987; Donchin and Coles, 1988; Coles and Rugg, 1996). Geometrical figures (CS) paired with corneal puffs of air (US) have increased peak-to-peak amplitudes from P100 to N180 and from N180 to P250 (Sugawara et al., 1977).

In the chemosensory modalities, both olfactory and gustatory USs have been paired with visual CSs. Neutral faces combined with either a pleasant or an unpleasant odor have increased the N100 peak of the VEP, and the aversive odor conditioning enhanced the Late Positive Complex (LPC; Hermann et al., 2000). After being paired with the aversive odor (fermented yeast), the neutral faces were rated as aversive, and the case therefore represents yet another example of plasticity in late VEP components related to evaluative conditioning. Another study paired colored geometrical figures (CS) with the taste of glucose and found that amplitudes of the CS-evoked P3 waves increased as the result of training (Franken et al., 2011). A recent first EEG-study of the effects of long-term image-flavor conditioning reported that N2-peak to P3-peak amplitudes were enhanced 1 day after pairing images with an appetitive flavor (Viemose et al., 2013). Visual evoked currents in the posterior part of the brain also increased. Further examples of sensory evoked potentials modified by conditioning were recently reviewed (Christoffersen and Schachtman, 2015).

The goal of the present study was to further elucidate the plasticity of VEPs induced by long-term visuo-gustatory conditioning. The first goal was to make a within-subject comparison between the effects of an appetitive and an aversive taste (US) on VEPs evoked by unfamiliar images (CSs). A second goal was to assess possible lateralization of learning-induced VEP plasticity. Third, the study aimed at examining a possible relationship between the magnitude of VEP plasticity and the perception of hedonic valence of the pleasant and unpleasant flavors. Although previous studies have documented correlations between the affective strength of a US and associative learning in humans (Buchanan and Lovallo, 2001; Cahill and Alkire, 2003; Cahill et al., 2003; Wittman et al., 2011), a correlation with the underlying neural plasticity has not yet been found. In contrast, animal investigations have reported correlations between the US affective strength and the magnitude of learning-induced neural plasticity such as long-term potentiation (LTP; Diamond et al., 2007; Wang et al., 2010; Lisman et al., 2011).

## Materials and Methods

### Subjects

Twenty-two healthy adult right handed subjects participated, age 20–41 years (mean ± SD: 29.6 ± 6.0). The subjects were instructed not to smoke, eat or drink anything but water 1 h before the experiments. Subjects were randomly allocated to one of two groups: a conditioned group (*n* = 12) that received training with images (CS) paired with one of two differently flavored apple juices (US) and a sham-trained control group (*n* = 10) stimulated with the images but not with US. Subjects were asked to blink only in the intervals between images during VEP-recording sessions. The study was exempt from ethical approval in accordance with the rules of the Danish Science Ethics Council. Reasons: the study was non-invasive, involved no drugs or harmful substances, induced no stress or pain.

### Apparatus

#### EEG-Recordings

One-hundred and eighteen EEG-channels were used (ANT Neuro, Enschede, Netherlands). Electrode cables were actively shielded (“Waveguard Cap”) and electrodes were placed according to the standard “5% system” (Oostenveld and Praamstra, 2001) using common average reference. Two electrode pairs placed above and below and at the outer edges of the eyes recorded EOGs and saccades. Sampling frequency was 512 Hz.

#### Visual Stimuli (CSs)

**Figure 1:**
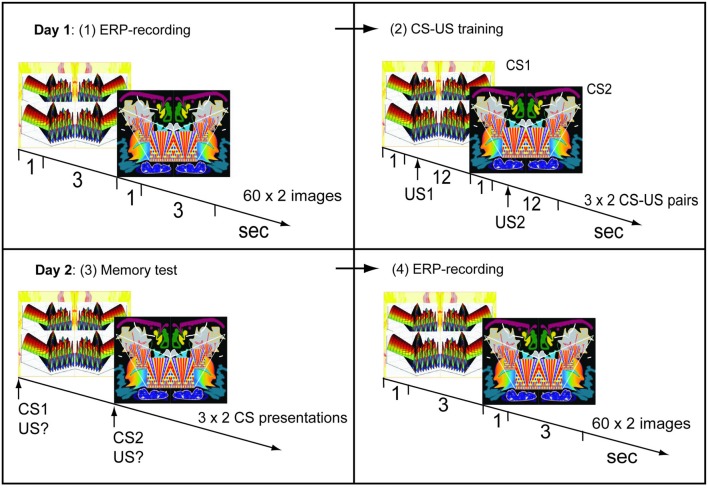
Experimental procedure. Day 1. (1) Two symmetrical and unfamiliar images were presented 60 times each in semirandom sequence. Visual evoked potentials (VEPs) were obtained for each image in this pre-training recording session. (2) Image-taste conditioning: one image (CS1) was paired with an appetitive juice, the other (CS2) with an aversive juice. Day 2. (3) Memory test: presentations of CS1 or CS2 cued a demanded description of the associated juice. (4) Post-training recording of VEPs using the same procedure as on day 1, before training.

Subjects were in a supine position with a monitor placed above their eyes. A fixation cross was placed in the middle of a rectangular monitor and images were observed under an angle from left to right of 56° and from top to bottom of 44°. The images were symmetrical along a vertical axis through the fixation cross (Figure 1). The timing of image presentations was controlled by a Matlab program (Mathworks, Natick, MA, USA), which also generated event-markers in the EEG-recordings at each stimulus onset.

#### Flavor Stimuli (USs)

Over a period of 2 s, 5 ml juice was delivered from a syringe into the mouth of the subject. A commercial clear apple juice (Rynkeby, Denmark) served as an appetitive US. An aversive version of the same juice was made by adding 8 g/L of glutamate, 0.256 g/L of ferrous-sulfate and 16 g/L of NaCl.

### Experimental Procedures

#### The Conditioned Group

##### EEG-recording before training on day 1

EEG-recordings were made during repeated presentations of two unfamiliar images (Figure 1). Each image was presented for 1 s 60 times and the two images were presented separately in semi-random sequence. The interstimulus interval was 3 s.

##### Training on day 1

After the EEG-recording, subjects received CS-US pairings (Figure 1). An image was presented for 1 s followed by injection of juice into the mouth beginning 1 s after the image had disappeared. In half of the subjects one of the images was followed by the appetitive juice and the other by the aversive one, while in the other half the opposite pairings were used. Three CS-US pairings were presented for each of the two types of juices. The two types of pairs were delivered in semi-random sequence and pairings were separated by 12 s.

##### Hedonic evaluation on day 1

After training, subjects were asked to evaluate their liking or disliking of the juices on a scale ranging from −5 to +5, representing respectively, the most unpleasant and most pleasant flavor imaginable.

##### Memory test on day 2

Twenty-four hours later, retention of conditioning was tested in the following way: an image was presented for 1 s and subjects were asked to verbally describe which of the two juices had been associated with the image. This was repeated three times for each of the two images (in semi-random sequence). Subsequent VEP-recordings were only performed if all six answers had been correct.

##### EEG-recording on day 2

An EEG-recording session was performed 1 day after the first using the same procedures as in the first session.

#### The Control Group

A sham-trained group was taken through the same procedures as the conditioned group except that, during training on day 1, the two CSs were presented without USs.

### Data Analysis

#### EEG Data

**Figure 2:**
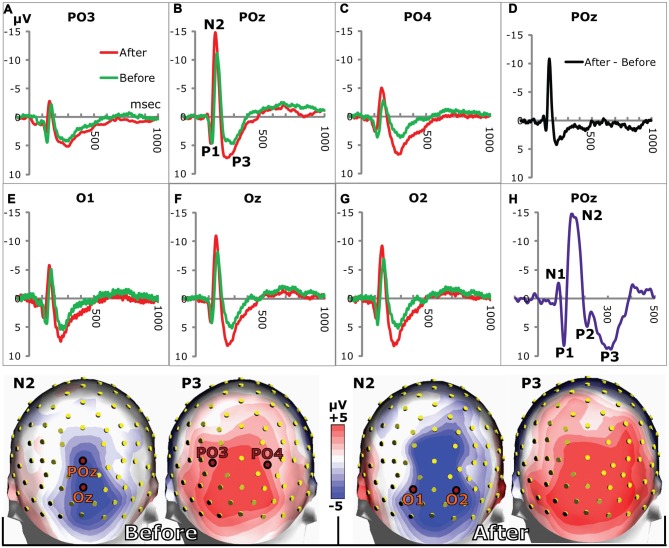
VEPs in posterior electrodes. **(A–C,E–G)** show grand average VEPs in the stated electrodes (marked in the headmodels at the bottom). The N2 and P3 peaks were larger on day 2 after training (red lines) compared to day 1 before training (green lines). **(D)** The difference between grand average VEPs (after training minus before) for POz illustrates the increases of N2 and P3. **(H)** VEP from a single subject showing all VEP-components from N1 to P3. Due to individual variations of VEPs, only P1, N2 and P3 prevailed in the grand averages. Bottom row: grand average head maps of posterior potential distributions at the N2 and P3 peaks before (left) and 24 h after (right) appetitive taste association.

**Figure 3:**
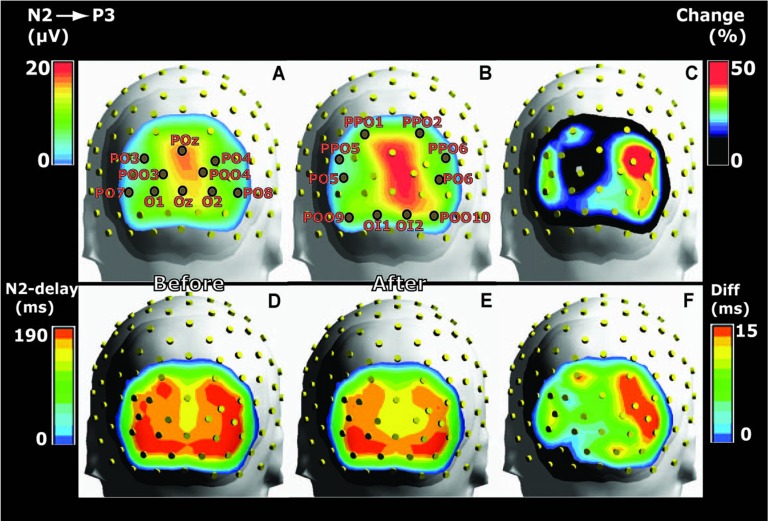
Grand average head maps of the posterior distributions of the potential differences between N2 and P3 peaks before and after training. **(A)** The distribution of N2-to-P3 amplitudes before training indicates a right side dominant response to the symmetrical images. **(B)** Amplitudes recorded 1 day after training are increased compared to panel **(A)**, and the right side dominant response persists. **(C)** Distribution of the average percentage change of N2-to-P3 amplitudes from before to after training. Percentages were calculated for each electrode on the basis of within-subject changes and averaged across subjects. The percentage map illustrates right side dominant learning-induced increases of amplitudes. **(D,E)** Posterior distributions of N2 peak delays before **(D)** and after training **(E)**. Delays were shorter along the midline where N2-P3 amplitudes were largest **(A,B)** compared to more lateral sites. The delays were reduced after conditioning. **(F)** The difference (Diff) between N2 peak delays before and after conditioning calculated within-subject for each posterior electrode and averaged across subjects. The distribution of delay plasticity reveals a right side dominance. All maps represent pooled results for appetitive and aversive conditioning.

EEG-data were analyzed with ASA Software (ANT Neuro, Enschede, Netherlands), EEGLAB (Swartz Center for Computational Neuroscience, University of California, San Diego, CA, USA) and custom made extensions to EEGLAB. Off-line band-pass filtering was made between 0.5 Hz and 30 Hz (36 db/octave). Artifacts from full or partial blinks, saccades and movements of the body or head were eliminated by rejecting trials in which they appeared. An initial artifact detection was made by automatic removal of trials containing artifacts that exceeded plus or minus 150 μV. Subsequently, the remaining trials were inspected and rejected if affected by smaller artifacts. The procedure reduced the 60 trials used before appetitive training on day 1 to an average of 53.3 trials, and to 53.4 after training. For the aversively associated images, the remaining numbers of trials were 55.7 on day 1 and 54.0 on day 2. The epoch used for averaging of VEPs ranged from 100 ms pre to 1000 ms post stimulus onset. Baseline correction was made using the mean potential of the pre-stimulus period. The potential differences between the N2 and P3 peaks (P3 peak value minus N2 peak value) were read for 20 posterior electrodes (shown in Figure 3): OI1, OI2, O1, Oz, O2, POO9, POO3, POO4, POO10, PO7, PO5, PO3, POz, PO4, PO6, PO8, PPO5, PPO1, PPO2, PPO6. This group of electrodes was selected as one that covered a coherent area over posterior visual cortices, and showed learning-induced changes of N2-to-P3 peak amplitudes. Grand average VEPs in the 20 electrodes were compared before and after training (Figure 2). Grand average VEPs were obtained after synchronization of the N2-peaks for each subject with the group mean N2 peak delay. This synchronization was used in order to avoid distortions of grand average N2 waves that would otherwise have been caused by individual differences in peak delays.

**Figure 4:**
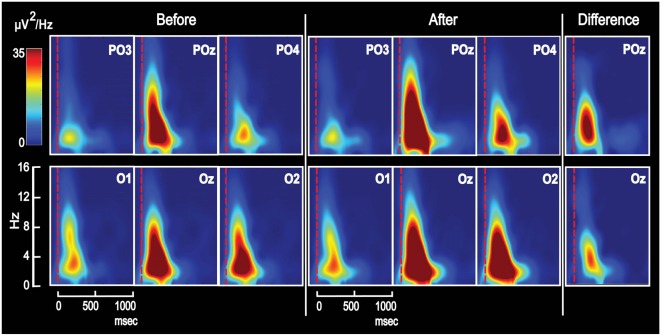
Grand average Time-Frequency-Power plots for the same six posterior electrodes as in Figure 2. The majority of VEP power appeared in the delta and theta frequency domains—both on day 1 for unassociated images and on day 2 after associative training. After training, power was enhanced, and the difference is seen to the right for POz and Oz. The figure shows results from appetitive training.

**Figure 5:**
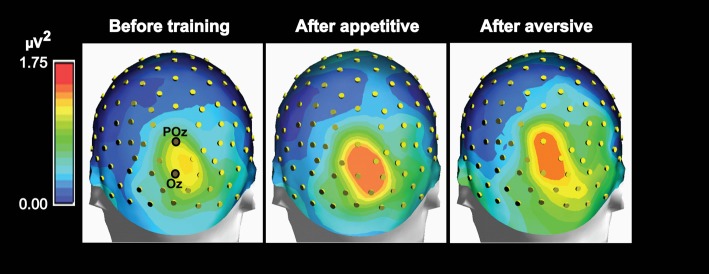
Maps of group mean posterior theta power topography before and 1 day after appetitive or aversive image-taste conditioning. Both the appetitive and the aversive CS-US pairings led to increased posterior power and both before and after conditioning, a right side dominant theta power is present in accordance with the lateralized N2-P3 amplitudes seen in Figure 3.

The temporal development of spectral power distribution for the VEP was calculated on the basis of fast fourier transformation (FFT; Figure 4). The VEP-epoch was extended at the left and right edges of the plot by one half of a sliding Hann window which covered 500 ms and moved with a step size of 2.75 ms. Head distribution maps for power in the theta frequency range of 4–8 Hz (Vernon et al., 2003; Babiloni et al., 2006; Kouijzer et al., 2009) were obtained from VEPs recorded in all 118 electrodes. Power was calculated after FFT using a 750 ms time window starting at image onset; and the power maps were averaged across subjects before and after training (Figure 5). The values of theta power were logged for each of the 20 posterior electrodes in each subject for statistical comparisons.

#### Regional Current Analysis

Calculations of current distributions in the brain were based on data from all 118 electrodes and on swLORETA (Palmero-Soler et al., 2007). Currents were calculated using a spatial resolution of 7 × 7 × 7 mm. This divided the brain into 4556 numbered voxels of 0.34 cm^3^. The Thalairach coordinates for each voxel were used to identify those that were placed inside of five left and five right hemisphere regions-of-interest. The regions were: (1) cuneus (visual areas 1 and 2 above the calcarine fissure); (2) lingual gyrus (LG) and the medial occipital gyrus (visual areas 1 and 2 below the calcarine fissure); (3) the inferior occipital gyrus; (4) posterior part of the inferior temporal gyrus (ITG; posterior to 50 mm behind the anterior commissure (AC)); and (5) the middle part of the ITG (between 10 and 50 mm posterior to AC). The stereotaxic atlas of Mai et al. (2008) was used for identification of the anatomical region to which each voxel belonged (Mai et al., 2008). The current in each voxel was compared before and after conditioning for each subject for statistical assessments of change. The regional currents stated in Table 1 represent the mean voxel current in each of the 10 regions—averaged across subjects. Currents were calculated at the peaks of both the N2 and the P3 waves and the peak-times were specified in each recording as the average of peak times for the 20 posterior electrodes. The peak current was obtained as the average over a period beginning two samples before and ending two samples after each peak. Finally, grand average current distribution maps were calculated across subjects for the whole brain before and after conditioning (Figure 7).

**Table 1:** Average visual evoked currents in voxels within five left and five right hemisphere posterior visual regions are shown before and after conditioning.

	N2							
	Left			Right			% Change	
Region	Before	After appetitive	After aversive	Before	After appetitive	After aversive	After appetitive	After aversive
(1) cuneus	16.6	27.6**	30.0**	13.3	24.4**	24.4**	103.3	122.0
(2) sub calc. fiss.	12.4	24.3**	18.1**	11.3	19.3**	16.4**	106.4	98.5
(3) IOG	13.1	20.4**	18.6**	14.0	21.5**	23.1**	130.0	109.8
(4) posterior ITG	9.0	14.9**	17.6**	7.2	14.3**	20.5**	140.2	177.0
(5) mid ITG	7.5	14.0**	9.4**	6.8	13.4**	18.1**	125.4	135.2
	**P3**							
	**Left**			**Right**			**% Change**	
	**Before**	**After appetitive**	**After aversive**	**Before**	**After appetitive**	**After aversive**	**After appetitive**	**After aversive**
(1) cuneus	11.7	23.8**	24.7**	10.2	17.8**	19.1**	171.5	222.6
(2) sub calc. fiss.	8.7	22.0**	18.1**	9.2	16.5**	12.8**	162.9	175.1
(3) IOG	9.0	20.3**	14.9**	10.5	15.9**	13.9**	160.6	143.0
(4) posterior ITG	7.8	13.7**	13.9**	6.9	16.0**	11.7**	128.6	116.2
(5) mid ITG	7.1	12.9**	15.4**	7.4	17.4**	12.9**	144.7	190.8

The currents were calculated on the basis of swLORETA performed at the peaks of N2 and P3 (further details in “Materials and Methods” Section). Currents increased significantly after conditioning in all 10 regions: **p < 0.01. % changes are stated for the left and right side regions combined. calc. fiss., calcarine fissure; IOG, inferior occipital gyrus; ITG, inferior temporal gyrus. Values obtained after training are highlighted in bold characters.

**Figure 6:**
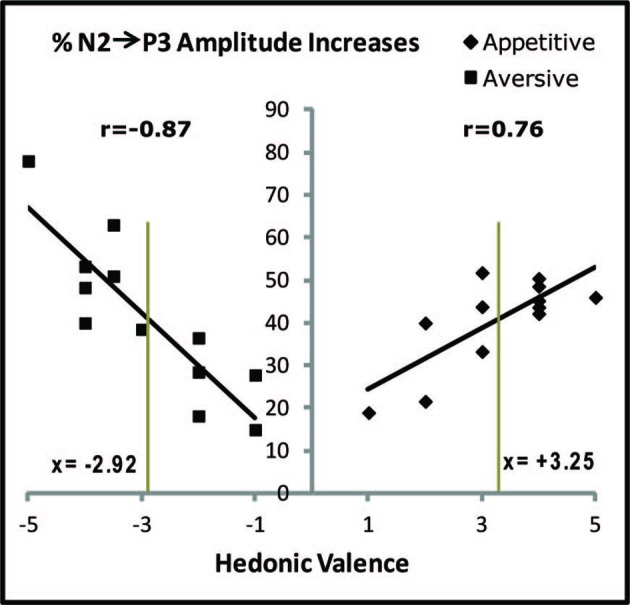
Correlations between individual hedonic scores and the percentage increases of N2-to-P3 amplitudes after conditioning. Percentage increases are plotted for each subject and represent the average percentage increases in the 20 posterior electrodes marked in Figure 3. In spite of the fact that only one appetitive and one aversive US was used, the individual hedonic scores covered the X-axis from 1 to 5 for the appetitive juice and from −1 to −5 for the aversive. Pearson correlation coefficients (r) are stated. The two vertical gray lines are placed at the group average positive and negative hedonic scores for the two juices (stated as x-values). The vertical gray lines cross the linear regression lines at nearly equal percentages (details in the text).

**Figure 7:**
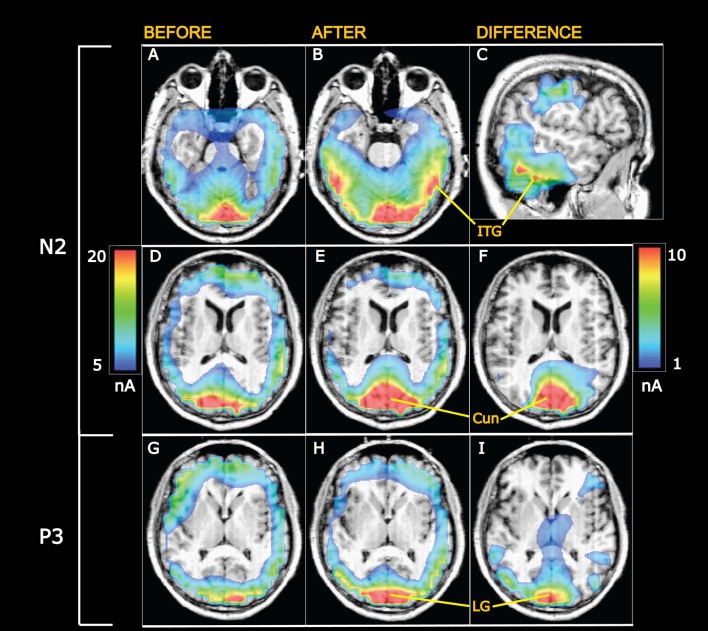
Grand average current density maps calculated from swLORETA. **(A–F)** Currents at the N2 peak. **(G–I)** Currents at the P3 peak. **(A,B)** Horisontal slices 20 mm ventral to the center of the anterior commissure (AC). After appetitive training **(B)** current densities are enhanced in the visual ventral stream going into the inferior temporal gyrus (ITG). **(C)** Sagittal slice 56.5 mm to the right of the AC showing the current difference (after minus before training) in the ITG. The scale to the right applies to the differential maps in the right column while the scale on the left is valid for the left and middle columns. **(D–F)** Horisontal slices 8 mm above the AC intersecting cuneus (Cun). The visual evoked currents increase from before **(D)** to after training **(E)**, and the difference is shown in **(F)**. **(G–I)** Currents at the P3 peak in horisontal sections 2 mm dorsal to AC at the level of the lingual gyrus (LG), showing increased visual evoked currents after conditioning.

#### Statistical Analysis

Data derived from the 20 posterior electrodes were used to test the statistical significance of changes in VEP amplitudes between recordings made before vs. after training. VEP amplitudes were submitted to a 2 × 20 analysis of variance (ANOVA) with session (pre and post training) and electrode as within-subject factors (the electrodes are listed above). A similar two factor ANOVA was used to compare values of VEP power derived from the same electrodes. In order to investigate laterality effects, VEP amplitudes were submitted to a 2 × 9 ANOVA with hemisphere (right and left) and electrode as within-subject factors (left electrodes: OI1, O1, POO9, POO3, PO7, PO5, PO3, PPO5 and PPO1 vs. their symmetrically placed right side counterparts). This was done separately before training, after appetitive and after aversive conditioning. Similar analysis was made for tests of lateralizations of power. Data from all 118 electrodes used to calculate voxel currents in the regions-of-interest (Table 1) were compared within-subjects before vs. after training using 2 × 10 session by region ANOVA for main effects of training. The significances of changes in individual regions were assessed from *post hoc* analysis (Fisher’s least significant difference). Further specifications regarding the applied statistical methods are described in the “Results” Section next to each statement of significance. Unless otherwise stated, average values are given with standard error of mean.

## Results

### Hedonic Evaluations

The average hedonic score for the apple juice was +3.25 ± 0.33, which was significantly above zero (*p* < 0.0001; *t*-Test vs. constant), and the juice was therefore rated as appetitive. The apple juice with added unpleasant tastants received an average score of −2.92 ± 0.37, significantly below zero (*p* < 0.0001) and was rated aversive. Although the two sets of scores were significantly different (*p* < 0.0001, unpaired *t*-Test), the absolute values of the two sets were not (*p* > 0.05). On the scale from −5 to +5, the two juices were therefore rated at distances from zero that were not significantly different—but with opposite polarities.

### EEG Data

#### Changes of the N2-P3 Waves Induced by Conditioning

Examples of grand average VEPs from six posterior electrodes are shown in Figure 2 before and after appetitive conditioning. All the examples show that the N2 and the P3 amplitudes were augmented after conditioning (Figures 2A–C,E–G). The difference between VEPs (after training minus before training) is shown for POz in Figure 2D, which illustrates enhancements of both N2 and P3 waves. All peaks from N1 to P3 are seen in Figure 2H, which was obtained from a single individual. In the grand averages for the whole subject group, only P1, N2 and P3 are seen, since individual differences in the N1 and P2 peaks eliminated them in the averaging across subjects. Quantitative measures for the learning-induced changes were obtained by logging the difference between N2 and P3 peak potentials (P3 peak minus N2 peak) for the 20 posterior electrodes seen in Figure 3. The group mean values for the N2-to-P3 peak differences in the posterior electrode group were 9.9 ± 0.3 μV before vs. 11.7 ± 0.4 μV after appetitive conditioning and 11.1 ± 0.4 μV after aversive conditioning. The increase of amplitudes induced by appetitive conditioning showed a significant main effect of training (*F*_(1,19)_ = 16.2; *p* < 0.0001; 2 × 20 session (pre and post training) by electrode ANOVA). A significant main effect of training was also found for aversive conditioning (*F*_(1,19)_ = 11.5; *p* < 0.001). There was no significant difference between the changes induced by appetitive vs. aversive conditioning: on average, appetitive conditioning increased the N2-to-P3 peak amplitudes in the 20 posterior electrodes by 27.6% ± 2.8% μV vs. 25.4% ± 2.8% for aversive training (*p* > 0.05; *t*-Test).

The N2 peak delays were shortened after conditioning (Figure 2). After appetitive training, the average delay in the 20 posterior electrodes across subjects changed from 172.1 ± 1.2 ms to 156.9 ± 1.2 ms while aversive conditioning reduced it to 162.4 ± 1.6 ms. The reduction after appetitive CS-US pairings gave a significant main effect of training (*F*_(1,19)_ = 11.0; *p* = 0.001, 2 × 20 session by electrode ANOVA) as did the aversive training (*F*_(1,19)_ = 19.5; *p* < 0.0001). The posterior distribution of peak delays and their change after conditioning is shown in Figures 3D–F.

In the sham-trained group which had no US presentations, the average N2-peak to P3-peak difference in the 20 posterior electrodes was 7.4 ± 0.4 μV on day 1 and 7.1 ± 0.3 μV on day 2 (*F*_(1,19)_ = 0.3; *p* > 0.05; 2 × 20 session by electrode ANOVA). The N2 peak delays were 166.9 ± 2.1 ms before and 165.2 ± 1.9 ms after sham training (*F*_(1,19)_ = 0.1; *p* > 0.05).

#### Lateralized VEPs and Effects of Conditioning

The largest N2-to-P3 amplitudes appeared in POz: 14.2 ± 1.5 μV before and 16.8 ± 2.0 μV after training (pooled results from appetitive and aversive training). However, in spite of the fact that both images were symmetrical across a vertical axis through the fixation point, the topographic distribution of group average N2-to-P3 amplitudes was not symmetrical in the two posterior visual hemispheres (Figures 3A,B). The figure shows a right side dominant response, confirmed quantitatively by comparing N2-P3 amplitudes in the nine left posterior electrodes with their nine right-side counterparts: The response to the two unfamiliar images presented before training (Figure 3A) gave an average N2-P3 amplitude in the nine left electrodes of 8.1 ± 0.3 μV vs. 10.1 ± 0.4 μV in the right side. A main effect of hemisphere was present (*F*_(1,8)_ = 22.4; *p* < 0.0001; 2 × 9 hemisphere by electrode ANOVA). After appetitive conditioning, the average left side amplitude was 9.3 ± 0.5 μV vs. 12.6 ± 0.5 μV in the right side. The difference represented a main effect of hemisphere (*F*_(1,8)_ = 26.6; *p* < 0.0001). After aversive training, the numbers were: 8.9 ± 0.5 μV for left and 12.0 ± 0.6 μV for right, and again the main effect of hemisphere was significant (*F*_(1,8)_ = 23.2; *p* < 0.0001). The asymmetry of N2-P3 amplitudes after conditioning is depicted in Figure 3B (collapsed data after appetitive and aversive training).

Next, it was tested whether conditioning had induced homogenously distributed augmentations of N2-P3 amplitudes, or whether the plasticity was not equally distributed among posterior electrodes. The topography of plasticity was assessed from the percentage increase of amplitudes (A) calculated for each posterior electrode in each subject ((A_after_ − A_before_) * 100/A_before_) and averaged across subjects (Figure 3C). The figure therefore shows the group average of within-subject changes of amplitudes rather than the change of the grand average values in Figures 3A,B. The figure illustrates that whereas the biggest N2-P3 amplitudes were found along the midline (Figures 3A,B), learning-induced increases were largest lateral to the midline; and the biggest augmentations appeared in the right side. Across both appetitive and aversive conditioning, the maximal average percentage increases occurred in PO4 (52%) followed by PPO6 (42%) and PO6 (39%). The average percentage increase in the nine posterior electrodes over the right hemisphere was 34.5 ± 3.1% vs. 25.1 ± 2.9% for the left side, and a main effect of hemisphere was observed (*F*_(1,8)_ = 5.1; *p* = 0.02; 2 × 9 hemisphere by electrode ANOVA).

The posterior distribution of N2 peak delays before training is shown in Figure 3D vs. after training in Figure 3E (pooled data for appetitive and aversive training). The figures illustrate that delays were shortest along the midline (where N2-P3 amplitudes were largest, Figures 3A,B). For example, before training POz had the shortest delay among the 20 posterior electrodes: 156.3 ± 5.0 ms vs. 144 ± 3.0 after training; in contrast, the lateral PO8 had the longest delays: 181.7 ± 4.7 ms before and 166.0 ± 5.3 ms after conditioning. The average within-subject reductions of N2 peak delays at the 20 posterior electrodes are shown in Figure 3F, which indicate a right side dominance of delay plasticity. The mean reduction in the nine right side posterior electrodes was 13.2 ± 1.6 ms vs. only 6.3 ± 1.6 ms in the left side, and a main effect of hemisphere was found (*F*_(1,8)_ = 9.3; *p* = 0.002; 2 × 9 hemisphere by electrode ANOVA).

#### Effects of Conditioning on Scalp Power Topography

In order to observe the temporal development of power spectra for the VEP and identify the frequency ranges in which conditioning changed power, plots of power as a function of time and frequency were obtained and averaged across subjects (Figure 4). The figure shows such time-frequency-power plots for the same six electrodes as in Figure 2. All electrodes revealed increased power in the theta and delta frequency domains after conditioning, and the difference (after − before) is visualized for POz and Oz. Further data on learning-induced changes of theta power were obtained from its scalp topography calculated from VEPs recorded in all 118 electrodes. Power was averaged from 0 ms to 750 ms into the VEP epoch. The resulting theta power topography is seen in Figure 5 before and after training. The figure indicates a right side dominant theta power in response to the symmetrical images—both before and after conditioning. This is in accordance with the lateralized distributions of N2-to-P3 peak amplitudes in Figures 3A,B. The statistical significance of lateralization was tested by comparing the mean power in the nine posterior left side electrodes to the mean of the nine right side counterparts. Before training, the average across subjects was 0.51 ± 0.04 μV^2^ for the left side vs. 0.86 ± 0.07 μV^2^ in the right side, and a main effect of hemisphere existed (*F*_(1,8)_ = 24.6; *p* < 0.001; 2 × 9 hemisphere by electrode ANOVA). After appetitive conditioning, the average for the left side was 0.89 ± 0.13 μV^2^ vs. 1.58 ± 0.18 μV^2^ for the right side (*F*_(1,8)_ = 10.4; *p* < 0.01) and after aversive training, the numbers were 0.63 ± 0.06 μV^2^ for left and 1.17 ± 0.10 μV^2^ for right (*F*_(1,8)_ = 20.8; *p* < 0.0001).

Quantifications of learning-induced increases of theta power were obtained for the 20 posterior electrodes in Figure 3. Across subjects, theta power was 0.74 ± 0.04 μV^2^ before training vs. 1.38 ± 0.11 μV^2^ after appetitive and 0.99 ± 0.06 μV^2^ after aversive training. Main effects of session (pre vs. post training) existed for both for the appetitive training (*F*_(1,19)_ = 18.2; *p* < 0.0001; 2 × 20 session by electrode ANOVA) and for the aversive (*F*_(1,19)_ = 23.7; *p* < 0.0001). The increases after the two types of training were not significantly different: a session by US type by electrode ANOVA found no effect of learning by US (*F*_(1,1)_ = 1.4; *p* > 0.05;). The latter result corroborated the observation described above of an absence of significant difference between effects of appetitive or aversive conditioning on N2-P3 amplitudes. In the sham trained group, there was no systematic increase of theta power from day 1 to day 2 (*F*_(1,19)_ = 0.04; *p* > 0.05; 2 × 20 session by electrode ANOVA). Maps of delta power topography showed learning-induced increases that were qualitatively analogous to the increases of theta power.

### Correlations between Hedonic US Evaluations and Plasticity of VEPs

Changes of N2-P3 amplitudes in each subject coupled with the individual hedonic scores for the USs allowed testing a possible correlation between VEP plasticity and hedonic perception. Such testing was made possible by the fact that the individual evaluations of liking or disliking of the USs were spread out over the entire hedonic scale from −5 to + 5 (X-axis in Figure 6). This spread was plotted against N2-P3 plasticity on the Y-axis (individual percentages of increase in average N2-P3 amplitudes for the 20 posterior electrodes). As a result of appetitive training, a positive Pearson correlation coefficient was found (*r*_(10)_ = 0.76; *p* = 0.004) while aversive training gave a negative coefficient (*r*_(10)_ = −0.87; *p* = 0.001). Long-term plasticity of the CS-induced VEPs was therefore correlated with the individual ratings of hedonic valence for the US.

The negative slope of the linear regression line in Figure 6 (aversive conditioning) had a value of −12.3 (Standard Error (SE) = 2.2), and the absolute value of this slope was greater than for the positive slope obtained after appetitive conditioning: 7.1; SE = 1.9. The significance of the difference in steepness was tested by converting the X-values for the aversive results to absolute values (moving the data on the left side of the Y-axis to the right side) before applying a one-way analysis of covariance (ANCOVA) which produced *p* = 0.09 for the difference of slopes, indicative of a trend towards stronger impact on plasticity for variations of aversive compared to appetitive US valence.

Next, the data for the regression lines were tested through a comparison with the conclusion obtained above that learning-induced N2-P3 percentage increases in the subject group as a whole were independent of US type. This result may be compared to percentage changes of N2-P3 amplitudes predicted by the regressions lines. For appetitive conditioning, the regression line was: *y* = 7.1*x* + 17.4 and for aversive: *y* = −12.3*x* + 5.5. When group mean hedonic scores are inserted (*x* = 3.25 for the appetitive US and *x* = −2.92 for the aversive; the values are marked as the two vertical gray lines in Figure 6), then the plasticity predicted from the appetitive regression line is 40.5% vs. 41.4% for the aversive. These near-equal percentage changes (at the crossings between the vertical lines and the regressions lines) are in agreement with the observation of non-significantly different group changes of N2-P3 amplitudes after conditioning with the two types of USs.

### Data for Cortical Current Distributions

Current distributions within the brain were compared before and after conditioning using swLORETA with a voxel size of 7 × 7 × 7 mm (details in “Materials and Methods” Section). Currents were calculated at the peaks of N2 and of P3. Subsequently, voxels (each voxel named by a number) were grouped in selected regions-of-interest. Five regions were selected in the early activated visual cortices and in the visual ventral stream as described in “Materials and Methods” Section and listed in Table 1. The average voxel-currents within each of these five left and five right side regions were found for each subject and group mean values are provided in Table 1 (nanoAmps). For currents at the N2 peak, a main effect of appetitive training was found (*F*_(1,9)_ = 176.1; *p* < 0.0001; 2 × 10 session by region ANOVA). *Post hoc* analysis found that the currents rose significantly after training in all five left and five right side regions with the levels of significance marked in Table 1. The percentage within-subject increases of currents were found in each of the five regions (average of left and right), and group average values are shown in Table 1. Visual evoked currents (VECs) at the N2 peak also revealed a significant increase after aversive training across the five left and five right regions (*F*_(1,9)_ = 194.8; *p* < 0.0001; 2 × 10 session by region ANOVA). *Post hoc* analysis found that the increases were significant in all 10 regions (levels of significance are marked in Table 1). The regional percentage increases of VECs after aversive conditioning are shown in Table 1 for left and right sides combined.

The data in Table 1 also show decremental VECs with increasing region no. A main effect of region was significant before training in the left hemisphere (*F*_(4,1027)_ = 18.3; *p* < 0.0001; one-way ANOVA) as well as in the right side (*F*_(4,1027)_ = 22.9; *p* < 0.0001). The decline was also present after appetitive learning in left (*F*_(4,1027)_ = 15.4; *p* < 0.0001) and right sides (*F*_(4,1027)_ = 15.7; *p* < 0.0001) as well as after aversive conditioning with *p* < 0.0001 for each side. In contrast to these declines of VECs along the five regions, the training-induced percentage increases of the VECs did not vary significantly across regions: Within-subject percentage increases of voxel-currents in the regions (left and right combined) were compared using one-way ANOVA, and no main effect of region was observed after neither appetitive nor aversive training (*p* > 0.05 in both cases). This observation is indicative of equal VEC plasticity in the five regions-of-interest.

For currents at the P3 peak, a main effect of appetitive training was observed (*F*_(1,9)_ = 144.2; *p* < 0.0001; 2 × 10 session by region ANOVA). *Post hoc* analysis revealed significant current increments after training in all 10 regions (Table 1). After aversive training, a main effect of training was also found for P3 peak currents (*F*_(1,9)_ = 335.4; *p* < 0.0001; 2 × 10 session by region ANOVA) and the levels of significance for current increments in each region are listed in Table 1. The percentage increases of VECs in the five regions (left and right combined) are shown in Table 1, and a one-way ANOVA across regions showed no significant main effect of region after neither appetitive nor aversive conditioning (*p* > 0.05).

Grand average current density maps are presented in Figure 7 for currents at the N2 and P3 peaks before and after training along with differential maps in the right column. The top differential map shows learning-induced increases of VECs at N2 along the ITG. The middle differential map visualizes the increases of N2 peak currents in a horizontal section through cuneus (Cun). Enhancements of VECs at the P3 peak are illustrated in the bottom differential map in a horizontal section at the level of the lingual gyrus (LG).

## Discussion

Identification of food-items and beverages based on visuo-gustatory associations are rapidly learned by primates, and also rapidly relearned in cases of counter-conditioning when the reinforcer changes its valence (Rolls, 2000; Stevenson et al., 2000). In spite of the essential role for human survival of the ability to visually identify edible items and their flavor, the dynamics of the underlying neurophysiological long-term plasticity is just beginning to be investigated. Recent work from our laboratory on the long-term effects of appetitive image-flavor conditioning (Viemose et al., 2013) was presently expanded by examination of plasticity induced by associations between unfamiliar, symmetrical images and either an appetitive or an aversive juice. The results showed that conditioning with both types of reinforcers augmented the amplitudes of the N2-P3 components. These changes appeared in the theta and delta frequency domains, and theta power head maps showed learning-induced increments over posterior visual areas 1 day after conditioning. The enhanced VEP waves were accompanied by increases of visual evoked currents in the ventral visual stream.

### VEP-Component Names and Functions

In order to facilitate comparisons between peaks described in this and other investigations, it may be pointed out that a negative peak with a delay between 100 ms and 200 ms has been called N1 by some authors (Mangun and Hillyard, 1991; Luck et al., 1994; Sauseng et al., 2005) rather than N2 as in the present work. The choice of N2 was motivated by the fact that an earlier negative peak (delay between 50 ms and 100 ms) has previously been observed before P1 (Roger and Galand, 1981; Aminoff and Goodin, 1994) and was also seen in some VEPs in the present study (Figure 2H). This early N1 was, however not frequent enough to appear in the grand average VEPs of Figure 2. The P3 of the grand average VEPs was occasionally preceded by a P2 (Figure 2H). However, in most subjects, the slope from N2 to P3 was so steep that the P2 either disappeared or only appeared as a small potential deflection between the two more prominent N2 and P3 peaks.

The N2 and P3 waves are late, so called “endogenous” components; a term that refers to the fact that their amplitudes are not exclusively determined by the features of external stimuli but are also governed by the internal states of the brain when the external stimulus is perceived. This involves emotional states (Begleiter and Platz, 1969a; Johnston et al., 1986; Mini et al., 1996; Naumann et al., 1997) as well as cognitive states including attention (Sutton et al., 1967; Rockstroh et al., 1989; Başar-Eroglu et al., 1992; Mangun and Hillyard, 1996; Pause and Krauel, 2000; Folstein and Van Petten, 2008). The roles of P3 in cognitive processing has been described in a “context updating theory” (Donchin and Coles, 1988) according to which, P3 is involved in cognitive updating from reference memory of information associated with a perceived stimulus (Ruchkin et al., 1988; Rösler and Heil, 1991; Naumann et al., 1992; Coles and Rugg, 1996; Skrandies, 1998). The present observations lend support for the theory since an increase of the P3 amplitude occurred as the CS-induced update changed from no association before training to taste-association after.

As an alternative to the interpretation of VEP-plasticity being related to conditioning, an effect of attention should be addressed. Variations in levels of attention have a strong impact on the amplitudes on endogenous wave amplitudes in sensory evoked potentials (Näätänen, 1992; Mangun and Hillyard, 1996). The present investigation used fixed inter-stimulus intervals which allowed for timed anticipation of image-presentations. If such anticipatory attention changed systematically between recording sessions on days 1 and 2, this could have affected the N2-P3 amplitudes. However, the sham trained group was subjected to the same repetition of recording sessions as the group that was trained with USs, and the sham group showed no significant change of the amplitudes. Consequently, it can be ruled out that systematic and unintended differences of attention between sessions 1 and 2 have contributed significantly to the observed N2-P3 changes.

Constant inter-stimulus intervals were used presently rather than randomly varying ones that are often used as a countermeasure against anticipatory EEG-activity such as “Stimulus Preceding Negativity” (SPN; Chwilla and Brunia, 1991; Brunia, 1993; Damen et al., 1996; Engdahl et al., 2007) or alpha wave suppression (Thut et al., 2006; Palva and Palva, 2007). The use of random variations were, however, found unnecessary in the present work, since preparatory experiments had revealed that no anticipatory activity occurred. This may have been due to the fact that the present EEG-recordings involved passive viewing without using the images as cues for active volitional operations, since such functions as cues are often preceded by anticipatory activity, as for instance “Contingent Negative Variations” (CNV; Walter et al., 1964; Green et al., 1970; Ruchkin et al., 1995; van Rijn et al., 2011).

### Images and Evoked Potentials

The images were designed with a triple purpose. First, it was desired to use unfamiliar images that were not associated with any flavor before training. Second, the images were complex in order to activate as many types of visual neurons as possible and generate large evoked potentials with good signal-to-noise ratios. The images activated both central and peripheral visual fields covering both foveal and extra-foveal areas. They also contained features that would excite both color and luminosity cells in the parvocellular ventral visual stream (Lueck et al., 1989; Zeki et al., 1991; Merigan and Maunsell, 1993; Shen et al., 1999; Wurtz and Kandel, 2000; Zeki, 2001; Reddy and Kanwisher, 2006; Fisch et al., 2009; Cardin et al., 2011). Third, the images were bilaterally symmetrical in order to allow examination of lateralization of the N2-to-P3 amplitudes and their plasticity (Figure 3).

In a previous examination of long-term visuo-gustatory conditioning in our laboratory, the images that were used as CS were asymmetrical (Viemose et al., 2013). This resulted in an observed left side dominant plasticity of N2-to-P3 amplitudes in contrast to the presently seen right side dominance of both N2-P3 amplitudes and their plasticity (Figure 3). Comparing the two investigations therefore demonstrates that lateralization of plasticity during visuo-gustatory conditioning is image-specific. In particular, images that activate hemifields symmetrically can lead to right side dominant plasticity.

### Regional Current Analysis

The observed enhancements of N2-P3 amplitudes after conditioning suggest learning-induced increments of visually evoked synaptic currents. This was tested using the “inverse problem” solution algorithm swLORETA (Pascual-Marqui et al., 1994, 2002; Palmero-Soler et al., 2007). Being a distributed source modeling method, current sources are identified as local current maxima within the current distribution maps generated by LORETA, and the accuracy of such source localization has been tested by comparisons with fMRI-scanning results (Vitacco et al., 2002; Mulert et al., 2004). Presently, the algorithm revealed that currents were elevated 1 day after training in the visual areas V1 and V2—both above and below the calcarine fissure (regions 1 and 2 in Table 1). These early activated areas (Machielsen et al., 2000; Vanni et al., 2001) project via the ventral streams into the inferior temporal gyri (Goodale and Milner, 1992; Milner and Goodale, 1998), in which regions 4 and 5 (Table 1) also showed statistically significant learning-induced increments of VECs.

Within the five selected regions-of-interest, the learning-induced percentage increments of VECs did not change significantly between regions. This was true for VECs both at the N2 peak and at the P3 peak. It is therefore indicated that all five regions contributed with near-equal plasticity to the learning process.

### Correlation between Hedonic Evaluations and VEP Plasticity

Even though the physical and chemical properties of the appetitive and aversive juices were not varied, subjects liked and disliked them to varying degrees as seen in the hedonic scores of Figure 6. Within-subject correlations of hedonic valence and percentage change of the N2-P3 complex revealed a positive correlation for the appetitive US and a negative one for the aversive US. The correlations therefore suggest that the physical/chemical properties of the US were not the primary determinants for neural plasticity; instead, it was the subjective perception of the US hedonic valence that served as a controlling factor. This is the first EEG-investigation that provides quantitative correlations between hedonic valence and learning-induced long-term plasticity in the human brain.

The correlations may be compared to results from animal studies. Here, correlations between US affective strength and learning have been described since early in the 20th century (Yerkes and Dodson, 1908; Yerkes, 1909; Teigen, 1994; Diamond et al., 2007), and information is available regarding an underlying relationship between US strength, dopamine release and late LTP (Wang et al., 2010; Lisman et al., 2011). In humans, a relation between formation of memory and the affective strength of a stimulus is also well established (Colegrove, 1899; Buchanan and Lovallo, 2001; Cahill and Alkire, 2003; Cahill et al., 2003). Brain structures such as the midbrain and hippocampus (Wittmann et al., 2005) as well as the amygdala (Canli et al., 2000; Hamann, 2001; Phelps, 2006; McGaugh, 2004) are involved in the coupling between emotion and memory formation. However, correlations between US hedonicity and human brain plasticity of CS-evoked sensory potentials are poorly understood and need further elucidation.

### Types of Involved Learning

The observed electrophysiological changes could involve either of two types of learning: (1) acquisition of an association that allowed the individual to *predict* the flavor when presented with the image; and (2) evaluative conditioning. In the memory test on the second experimental day, subjects correctly described the identity of juice linked to the presented images, proving that predictive association between image and flavor-identity was present. Furthermore, due to the hedonic qualities of the two flavors, evaluative conditioning may also have been a driver for the observed plasticity. Further investigations are needed to make clear distinctions between the electrophysiological correlates of the purely predictive and the hedonic associations.

The present investigation was not concerned with the novelty or familiarity of the flavors that served as USs. Instead, the focus was placed on gaining fundamental information on human brain VEP plasticity after gustatory conditioning. The possible impact of US familiarity on VEP plasticity is therefore unknown at present. It may be considered possible that the familiarity of the apple juice could have inhibited conditioning through the “US pre-exposure effect” (Randich and LoLordo, 1979; Randich, 1981). However, a previous study from our laboratory also recorded potentiation of the N2-P3 waves after conditioning with unfamiliar appetitive yogurt flavors, in contrast to the presently used familiar apple juice. Therefore, the degree of familiarity with the appetitive US was not a critical determinant for N2-P3 potentiation.

### Neural Mechanisms Behind N2-P3 Plasticity

Regarding a synaptic background for plasticity of sensory potentials induced by conditioning, an extensive literature from rodent studies has shown the involvement of “LTP” (Martin and Morris, 2002; Muller et al., 2002; Morris et al., 2003; Pastalkova et al., 2006; Barki-Harrington et al., 2009; Benito and Barco, 2010; Wang et al., 2010; Lisman et al., 2011). In monkeys, LTP has been observed for instance in the hippocampus (Urban et al., 1996) and infero-temporal cortex (Murayama et al., 1997), and in the human brain, evidence for LTP is available for the temporal cortex (Chen et al., 1996; Cooke and Bliss, 2006; Hoogendam et al., 2010), the hippocampus (Beck et al., 2000), motor cortex (Stefan et al., 2000; McDonnell et al., 2007), auditory cortex (Clapp et al., 2005) and also the visual cortex (Teyler et al., 2005). However, although LTP may exist in the human brain, it has not yet been causally linked with conditioning. Enhancements of synaptic currents are a characteristic feature of the LTP process (O’Connor et al., 1995; Harney et al., 2008; Rebola et al., 2010), a fact that could explain the presently observed enhanced VECs in the five visual regions of Table 1. Such increases could, however, also have been caused by enhanced synchronization of population post-synaptic potentials.

Further indications of LTP-involvement in N2-P3 plasticity are provided by reports that have: (1) correlated human hippocampal activity with endogenous components such as N2 and P3 (Halgren et al., 1980; Okada et al., 1983); (2) convincingly established a role for human hippocampal activity in some types of non-spatial associations (Cave and Squire, 1991; Henke et al., 1997, 1999); and (3) shown that animal hippocampal LTP has an central role in conditioning (Martin and Morris, 2002; Muller et al., 2002; Morris, 2003; Malenka and Bear, 2004). Clearly, more evidence for a causal link between LTP and human associative learning is needed.

## Conclusion

The neurophysiology of visuo-gustatory memories has only been sparingly investigated although these memories form the basis of most food choice behavior. Previous findings of modified human VEPs after appetitive long-term visuo-gustatory conditioning (Viemose et al., 2013) were presently confirmed and expanded by the inclusion of aversive flavor conditioning. It was found that VEP waves over posterior visual cortex areas were potentiated after conditioning with both types USs. A map of the posterior distribution of plasticity showed a right hemispheric dominance of plasticity although the stimulations of the two visual hemifields were symmetrical. Visual evoked currents increased after conditioning in all investigated regions from visual areas 1 and 2 and along the visual ventral stream. A correlation was observed between human learning-induced VEP plasticity and subjective evaluations of the US hedonic valence.

## Author Contributions

GRJC: planned the experiments and served as experimental and theoretical tutor for two co-authors: PhD student IV and Master’s student CL, performed all data analyses, produced manuscript and figures. JLL: generated Matlab programs used in some of the data analyses. PM and WLPB: formal supervisors for PhD and Master’s students, provided laboratory facilities. TRS: provided information on human associative memory and made important modifications of the manuscript. CL: performed some of the experiments. IV: performed most of the experiments.

## Conflict of Interest Statement

The authors declare that the research was conducted in the absence of any commercial or financial relationships that could be construed as a potential conflict of interest.
